# Combined use of magnetic microbeads for endothelial cell isolation and enhanced cell engraftment in myocardial repair

**DOI:** 10.7150/thno.75871

**Published:** 2023-02-05

**Authors:** Christian Biederbick, Jan C. Heinemann, Sarah Rieck, Florian Winkler, Annika Ottersbach, Miriam Schiffer, Georg D. Duerr, Dietmar Eberbeck, Michael Hesse, Wilhelm Röll, Daniela Wenzel

**Affiliations:** 1 Institute of Physiology I, Life&Brain Center, Medical Faculty, University of Bonn, Bonn, Germany; 2 Department of Cardiac Surgery, Medical Faculty, University of Bonn, Bonn, Germany; 3 Department of Cardiovascular Surgery, University Medical Center Mainz, Johannes Gutenberg University, Mainz, Germany; 4 Physikalisch-Technische Bundesanstalt (PTB), Berlin, Germany; 5 Institute of Physiology, Department of Systems Physiology, Medical Faculty, Ruhr University of Bochum, Bochum, Germany

**Keywords:** magnetic microbeads, cell therapy, cell engraftment, myocardial repair, endothelial cells

## Abstract

**Background:** The regenerative potential of the heart after injury is limited. Therefore, cell replacement strategies have been developed. However, the engraftment of transplanted cells in the myocardium is very inefficient. In addition, the use of heterogeneous cell populations precludes the reproducibility of the outcome.

**Methods:** To address both issues, in this proof of principle study, we applied magnetic microbeads for combined isolation of eGFP^+^ embryonic cardiac endothelial cells (CECs) by antigen-specific magnet-associated cell sorting (MACS) and improved engraftment of these cells in myocardial infarction by magnetic fields.

**Results:** MACS provided CECs of high purity decorated with magnetic microbeads. *In vitro* experiments revealed that the angiogenic potential of microbead-labeled CECs was preserved and the magnetic moment of the cells was strong enough for site-specific positioning by a magnetic field. After myocardial infarction in mice, intramyocardial CEC injection in the presence of a magnet resulted in a strong improvement of cell engraftment and eGFP^+^ vascular network formation in the hearts. Hemodynamic and morphometric analysis demonstrated augmented heart function and reduced infarct size only when a magnetic field was applied.

**Conclusion:** Thus, the combined use of magnetic microbeads for cell isolation and enhanced cell engraftment in the presence of a magnetic field is a powerful approach to improve cell transplantation strategies in the heart.

## Introduction

Myocardial infarction resulting in heart failure is the leading cause of death worldwide 1. To restore myocardial function cell transplantation approaches with bone marrow cells, endothelial cells, cardiomyocytes or their precursors have been developed and some of them showed promising results 2,3. However, the number of cells engrafting into the infarct area is extremely limited and more than 90% of the cells injected are lost over time 4,5. Most likely, this can be explained by a combination of several factors such as leakage from the injection site, cell death, activation of immune responses, low oxygen tension and weak cell- matrix interaction 4,5. Therefore, cell transplantation strategies are currently very inefficient, which may be one of the reasons why they are not applied in routine clinical therapy. In one of our recent studies, we could show that short- and long-term engraftment of embryonic and embryonic stem cell-derived cardiomyocytes into the injured heart can be strongly improved when they are loaded with magnetic nanoparticles (MNPs) and cell injection is performed in the presence of a magnetic field 6. However, for efficient MNP loading the uptake of magnetic material into the cells during culture is required 7-9. Even when non-toxic MNP concentrations are used it cannot be completely excluded that cell function as well as the regenerative potential of the cells is compromised by intracellular accumulation of MNPs and cultivation of the cells. Many stem or progenitor cells that are promising cell populations for heart repair have to be isolated via specific cell surface markers by magnet-associated cell sorting (MACS) 10-13. Therefore, in the current study, we take advantage of these magnetic microbeads to combine cell isolation and magnetization of the cells. In a proof of principle approach, we expose MAC-sorted microbead-labeled eGFP^+^ endothelial cells to a magnetic field during and shortly after cell injection into myocardial infarction. Our data demonstrate that this leads to an enhanced engraftment of the cells resulting in neovessel formation, reduction of infarct size and improvement of cardiac function.

## Methods

### Isolation of embryonic and adult cardiac endothelial cells (CECs)

To obtain eGFP^+^ CECs the flt-1/eGFP 14 or the PECAM/eGFP 14 mouse line was used. Embryonic CECs were derived from embryos at stage E12.5-E16.5. Adult CECs were isolated from adult mice (8-15 weeks). For isolation of embryonic CECs embryonic hearts were excised and atria were removed. Ventricles from about 10 embryos were pooled. For isolation of adult CECs, adult hearts were harvested and perfused with PBS via the aorta, then the atria were removed. Ventricles of embryonic or adult hearts were minced and transferred to 1 ml pre-warmed enzyme cocktail containing 0.25 mg ml^-1^ Liberase TL Research Grade (Roche, Manheim, Germany) and 20 U ml^-1^ DNase I (Sigma-Aldrich, Steinheim, Germany) in sterile-filtered HBSS buffer (+Ca^2+^/Mg^2+^) supplemented with 10 mM HEPES in (Gibco/Invitrogen). Tissues were incubated at 37°C and centrifuged at 900 rpm for 20 min. After that the cell solution was transferred to 8 ml of ice-cold IMDM culture medium supplemented with 20 % fetal calf serum (FCS, Gibco/Invitrogen) while remaining heart tissue was exposed to a second digestion step using fresh enzymes. Finally, cell solutions were pooled and passed twice through a 40 µm nylon mesh filter (BD Bioscience, Heidelberg, Germany) to exclude non-digested tissue and debris. The cell solution was centrifuged (300 g) for 10 min at 4°C and the cell pellet was resuspended in MACS-buffer containing 2 mM EDTA and 0.5 % bovine serum albumin (BSA) in PBS (all from Gibco/Invitrogen). For MACS cells (about 200,000 embryonic CECs, 400,000-800,000 adult CECs) were labeled with anti-CD31-microbeads (1:10, Miltenyi Biotech, Bergisch Gladbach, Germany) for 15 min at 8°C. After two washing steps using MACS-buffer, labeled embryonic CECs were transferred to a MACS-MS-column (Miltenyi Biotech), for isolation of adult CECs a MACS-LS-column (Miltenyi Biotech) was used.

### Flow cytometry

To determine the purification efficiency of MACsorted CECs, flow cytometry of cell isolations was performed directly after MACS. Therefore about 1x10^6^ cells from (1) single cell suspensions directly after enzymatic digestion without MACS (pre-sort fraction), (2) single cell suspension after MACS from flow through (post-sort negative-fraction) and (3) single cell suspension after MACS adherent to the MACS column (post-sort positive-fraction) were taken, centrifuged at 300 g for 10 min at 4°C and resuspended in ice cold fluorescence-activated cell sorting-(FACS)-buffer consisting of 2 mM EDTA and 1 % FCS in PBS. To prevent unspecific binding of antibodies, all samples were incubated with mouse Fc block (BD Bioscience) for 5 min. Afterwards all samples were labeled with anti-CD31-PE antibody (1:1000, BD Bioscience) for 30 min at 8°C. To exclude dead cells from analysis, the samples were incubated with 1 μg ml^-1^ Hoechst-33258 (BD Pharmingen) shortly before flow cytometry analysis. Isotype controls were generated to exclude unspecific binding. Finally, all samples were analyzed using a CyFlow cytometer (Partec, Goerlitz, Germany) in combination with FlowJo software (Tree Star Inc., Ashland, USA). For embryonic CEC injections into hearts with cryoinfarctions, only preparations with > 85 % purity of CECs were used.

### Cultivation of embryonic and adult CECs

To compare the growth potential of embryonic and adult CECs 100,000 or 200,000 cells/well were plated directly after MACS on gelatine-coated glass coverslips in a 24-well plate in IMDM culture medium supplemented with 20 % FCS. Cells were fixated with 4 % paraformaldehyde (PFA, Merck, Darmstadt, Germany) after one and 7 days. To analyze sprout formation in embryonic CECs, approximately 0.2-0.5x10^6^ total cells were plated on Biocoat Matrigel® Matrix (BD Pharmingen) coated glass coverslips and cultivated in 24-wells in 1 ml of IMDM supplemented with 20 % FCS, 1 % penicillin/streptomycin solution and 0.1 % beta-mercaptoethanol (all from Gibco/Invitrogen) for 14 days.

### QPCR analysis

To analyze gene expression of CD31, flt-1 and flk-1 total RNA was isolated using Pure Link RNAeasy Kit® (Life Technologies). 2 μg of total RNA was used to generate cDNA using the High Capacity cDNA Transcription Kit® (Life Technologies, Darmstadt, Germany) as described by the manufacturer. QPCR was performed using pre-designed Taqman® assays (CD31:Mm01242584_m1, flk1: Mm01222421_m1, flt1: Mm00438980_m1) and GeneExpression Master Mix© (all from Life Technologies) in accordance with the manufacturer`s protocol. Gene expression levels were measured on an ABI Prism 7900HT Sequence Detection System in combination with SDS2.4 Software (Life Technologies). Gene expression of target genes (Ct) was normalized to gene expression of the housekeeper 18S rRNA (=ΔCt) and to the mean of the pre-sort population using the ΔΔCt-method as described before 15.

For the analysis of ephrin B2 (Efnb2), eph receptor B4 (Ephb4), endomucin (Emcn) and apelin (Apln) total RNA was isolated using RNeasy Plus Micro Kit (Qiagen). CDNA synthesis was performed with SuperScript^TM^ VILO^TM^ cDNA synthesis kit (Invitrogen/Thermo Fisher Scientific) according to the manufacturer's instructions. For qPCR Sybr Green PCR Kit (Qiagen) and the following QuantiTect Primer assays (Qiagen) were used: QT00095431 (Efnb2), QT00095431 (EphB4), QT00117082 (Emcn), QT00111762 (Apln), QT01036875 (18S rRNA). Gene expression levels were determined with a CFX Opus 96 Real Time PCR System (BioRad) and the corresponding CFX Maestro software (BioRad). Relative gene expression was calculated with the ΔCt method.

### Magnetic particle spectroscopy (MPS)

MPS measurements were performed as described before 9. The nonlinear part of magnetic bead magnetization was determined with a Magnetic Particle Spectrometer (Bruker BioSpin GmbH, Germany). The samples were exposed to a strong alternating field with an amplitude of μ0H = 24 mT and a frequency of f0 = 25 kHz. Then, the nonlinear part of magnetization of magnetic microbeads produces higher harmonics with amplitudes Ak and frequencies fk =k f0 which can be separated from the signal contribution of the base frequency f0 by high pass filtering. Thus, the signal is very specific for magnetic microbeads because components of diamagnetic and paramagnetic parts of the samples (e.g. tissue, cell medium, blood) do not contribute to Ak with k>1, as they have a linear magnetization behavior in the present field range. In order to quantify the magnetic microbeads` iron, 100 μl of the cell suspensions (200,000 cells) as well as a reference sample containing a known amount of magnetic microbeads was measured by the MP spectrometer. For quantification we used the two strongest amplitudes A3 and A5. We calculated the amount of iron within the sample according to m(Fe) = 1/2 mRef(Fe) (A3/A3,Ref+A5/A5,Ref). The measurement uncertainty of the individual samples was estimated by the difference according to u(mFe)=1/2 mFe,ref [(u(A3)/A3,ref)2+(u(A5)/A5,ref)2+(A3/A3,ref-A5/A5,ref)2/4]1/2. Finally, the total amount of iron was divided by the number of cells per sample to obtain the amount of iron per cell.

### Quasistatic magnetization M(H)

The M(H) curves of the fluid magnetic bead dispersions were measured using a commercial susceptometer (MPMS XL5, Quantum Design) which works with highly sensitive SQUID sensors. The samples were filled in polycarbonate capsules which in turn were fixed within a straw in order to center the samples inside the pickup coil system. Prior to the measurement, an empty capsule was measured, the signal of which was then subtracted from the data yielding the signal of the dispersion. Finally, the diamagnetic contribution of the dispersion medium (water) was subtracted from the data yielding the M(H)-curve of the magnetic microbeads. The M(H) curves of the bead-labeled cells were scaled to the reference curve of the applied microbeads with the scaling factor k=MRef(18 kA m^-1^)/M(18 kA m^-1^).

### Local positioning of magnetically labeled eGFP^+^ CECs *in vitro*

For local positioning experiments 200,000 eGFP^+^ CECs were seeded on cover slips in 24-well plates coated with 0.1 % gelatine solution directly after MACS. The 24-well plate was placed on a shaker and a rod magnet (30 mm diameter) with a 1 mm soft iron tip (~1.5 T, Zentralinstitut für Medizintechnik, TU München, Germany) was positioned underneath. The whole setup was agitated for 1 h at 125 rpm. After another hour without shaking the magnet was removed and the cultures were kept at 37°C in a humidified atmosphere of 5 % CO_2_. Pictures of native eGFP expression were acquired at d1 in culture and d10 after fixation and immunostaining with an Axiovert 200M microscope (Carl Zeiss, Oberkochen, Germany). Quantification of single eGFP^+^ cells, branching points and vascular loops was performed using WCIF Image J software (National Institute of Mental Health, Bethesda, USA). Hereby, the number of events was counted in predefined annular areas with a fixed radial distance from the center where the magnet was placed during plating*.*

### Animal experiments

All mouse experiments were performed in accordance with the ARRIVE guidelines, the guidelines of the German law of protection of animal life and approved by the local government authorities (Landesamt für Natur und Verbraucherschutz Nordrhein-Westfalen, NRW, Germany).

### Intramyocardial injection of MACsorted embryonic CECs and magnetic targeting

Transmural cryoinfarctions were generated in female CD1 mice at the anterolateral left ventricular wall under general anesthesia. For analgesia, 5 mg kg^-1^ s.c. carprofen was used until d3 after surgery. For cryoinfarction induction in CD1 mice a liquid nitrogen-cooled copper rod (3.5 mm diameter) was applied as described earlier 6. MACsorted embryonic CECs were transplanted into the center of the injury by a single intramyocardial injection with a 10 µl Hamilton syringe equipped with a 29G insulin needle. Either embryonic CECs + magnet (200,000 MACsorted cells re-suspended in 5 µl culture medium with superimposition (5 mm distance) of a 1.3 T bar magnet during and 10 min after injection) or embryonic CECs - magnet (200,000 MACsorted cells re-suspended in 5 µl culture medium without magnet application) were injected. The gradient of the magnetic flux density of the magnet was calculated by finite element calculations with the AC/DC module of the Comsol Multiphysics 4.3 package (Comsol Multiphysics GmbH, Göttingen, Germany). Control mice were injected with the same volume of medium but with no cells. Recipients were immunosuppressed by daily intraperitoneal injections of cyclosporin A (20 mg kg^-1^; Novartis).

### Hemodynamic analysis of left heart function

Two weeks after cell or control injection in mice, echocardiographic analysis and left ventricular pressure-volume catheter analysis were performed under inhalative anesthesia (isoflurane 1.0-1.5 vol.%). Echocardiographic analysis was performed using a HDI-5000 ultrasound system in combination with the linear array transducer CL15-7 (both from ATL-Phillips, Oceanside, CA, USA) working at 15 MHz and providing frame rates up to 284 Hz as described previously 16. Briefly, 2D-guided M-mode data were acquired in the parasternal short-axis at the level of the papillary muscle. Both fractional shortening (FS) as a global parameter of anterior left ventricular function as well as anterior wall thickening (AWT) as a regional parameter of left ventricular function (AWT = (AWsyst-AWdiast)/Awdiast x 100) 17 were recorded and analyzed. For left ventricular catheterization a 1.4-French pressure-conductance catheter (Millar Instruments, Houston, USA) was inserted retrogradely via the right carotid artery and advanced into the left ventricle. Data were continuously recorded for at least 5 min and ejection fraction (EF) and stroke volume (SV) were determined using LabChart® Pro software (ADInstruments, Spechbach, Germany).

### Histology and immunohistochemistry

After functional analysis, mice were sacrificed by cervical dislocation and hearts were excised and imaged by a fluorescence stereomicroscope (Axio Zoom V16, Carl Zeiss MicroImaging GmbH, Göttingen, Germany). Hearts were cannulated via the ascending aorta followed by perfusion with PBS and PFA solution (4%) and then fixated overnight at 4°C. Then, hearts were incubated in 20 % sucrose solution in PBS and cryopreserved in Tissue Tek O.C.T. compound (Sakura Finetek Zoeterwoude, Netherlands). Immunohistochemistry was exerted as described recently 18. Briefly, 10 µm cryosections were generated with a cryotome CM 3050S (Leica, Wetzlar, Germany). Cell engraftment was assessed by native eGFP of embryonic CECs. For fluorescence immunostainings sections were permeabilized with 0.1 % Tween-20 (Sigma-Aldrich) and blocked with 5 % donkey serum (Jackson ImmunoResearch, Suffolk, UK) for 1 h. Then, heart sections were incubated with anti-CD31 (1:400, BD Biosciences, Heidelberg, Germany) overnight at 8°C. After washing, secondary antibody donkey-anti-rat-Cy3 (1:400, Jackson ImmunoResearch) was applied for 1 h. Nuclei were stained using Hoechst 33342 (1:1000, BD Bioscience). For Masson trichrome staining of heart sections the Trichrome Stain kit (ab150686, abcam) was used. For staining of fixated cells additional primary antibodies were used: anti-alpha-smooth muscle actin (αSMAC, 1:800, Sigma-Aldrich), anti-CD45 (1:1000, Chemicon, Hampshire, GB), anti-alpha actinin (αAct, 1:400, Sigma-Aldrich) and anti-Ki-67 (1:200, abcam #15580), as secondary antibodies donkey-anti-rat-Cy3 and donkey-anti-mouse-Cy3 (both Jackson ImmunoResearch) were applied. For the staining of apoptotic cells after permeabilization a TUNEL-reaction mix (*In situ* death detection kit, Roche, #12156792910) consisting of 450 µl labeling solution and 50 µl enzyme solution per slide was used. After one hour at 37°C in the dark, the slides were washed with PBS three times and subsequently stained with Hoechst 33342. For visualization of MACS microbeads after cell isolation prussian blue staining was performed. Therefore, directly after sorting200,000 MACsorted cells were applied to a well of a 24-well plate and enriched in the center of the well by placing a magnetic tip underneath for 1 h 19. Then, cells were cultivated for additional 5 h. Cells were incubated with a staining solution containing 5% potassium ferricyanide II solution and 5 % hydrochloric acid (1:1) for 20 min. Subsequently, the cells were counterstained with eosin solution for 5 min. For analysis of native eGFP fluorescence and immunofluorescence in sections an Axiovert 200M microscopy system equipped with an Apotome was used. For analysis of prussian blue staining an AxioStar plus microscope was applied. Images were aquired with the AxioVision software (Carl Zeiss, MicroImaging GmbH).

### Quantitative morphometry

For calculation of the total amount of engrafted eGFP^+^ cells fluorescence pictures of up to 18 sections per heart at a distance of 100 µm were generated with a stereomicroscope (Axio Zoom V16, Car Zeiss MicroImaging GmbH, Göttingen, Germany). Then, eGFP^+^/Hoechst^+^ cells were counted manually. Cell numbers were extrapolated to the total amount of heart sections correcting for overlap based on the average nuclear size. Fluorescence images of 8-14 unstained sections per heart covering the infarct were obtained to calculate infarct volume and epicardial areas. The infarct zone was identified based on the lower autofluorescence signal of the tissue. Infarct areas and epicardial surfaces lengths were traced manually in digital images, quantified by a measuring tool and then extrapolated to the total infarct volume. For the calculation of LV wall thickness, pictures of 6-12 unstained sections at a distance of about 300 µm per heart were used. LV wall thickness was measured at 5 different positions per section: the mid-infarcted area, both border zones and between mid-infarction and border zones using fluorescence pictures as described above. Then, the mean of the 5 positions per section was calculated followed by the average diameter of all measured sections.

### Microscopy

For documentation of cell engraftment in heart slices and for evaluation of planimetric parameters regarding to myocardial infarction, fluorescence pictures of representative, unstained or Hoechst-stained slices were recorded using a macroscope in combination with ZEN2011® software (both from Zeiss, Jena, Germany).

### RNA-seq analysis

For RNA-seq experiments DNA-free total RNA was isolated from MACsorted embryonic (n = 5) or adult CECs (n = 6) (positive fraction) as well as the remainder of the embryonic hearts (n = 5) (negative fraction) using the RNeasy Kit (Qiagen) including on-column DNAse digestion. RNA quality was analyzed by an Agilent Bioanalyzer (Agilent). For library preparation the Trio RNA-Seq Library Preparation kit for mouse (TECAN) was used, starting with 50 ng of total RNA. Thirteen PCR cycles were used for library amplification and libraries with an average fragment size of 380 bp were sequenced on a NextSeq 500 in paired-end mode (75 bp, Illumina). For bioinformatic analysis we used the Galaxy platform (Freiburg Galaxy Project 20). RNA sequencing reads were mapped using RNA STAR 21 followed by counting reads per gene by using featureCounts 22. Differentially expressed genes were identified by DESeq2 23. For data visualization, normalization and cluster analysis heatmap2 and Volcano plot (Freiburg Galaxy Project 20) was used. Gene ontology (GO) analysis of the 300 top up- and downregulated genes was performed with ClueGO using the GO-term database with the sub-ontology “biological processes”. Gene Set Enrichment Analysis (GSEA) was performed by using the pre-ranked gene list method implemented in the java-based GSEA program and the C2 and C5 curated molecular signature database both provided by the BROAD institute (http://www.broadinstitute.org/gsea/index.jsp).

### Statistical analysis

All statistical evaluations were done using Prism 5 (GraphPad, San Diego, USA). Data are presented as mean +/- SEM. To compare differences between more than two groups one-way ANOVA with Tukey`s post hoc test was performed. Additionally, unpaired t-test was used to compare two groups. P values p < 0.05 were considered significant.

## Results

### Isolation of CECs by MACS and *in vitro* characterization of the cells

To identify a suitable endothelial cell type for magnet-guided cell injection into myocardial cryoinfarction we first isolated embryonic (Figure S1A) and adult (Figure S1B) hearts of the flt1/eGFP mouse line; in this mouse line endothelial cells are labeled with the fluorescence reporter eGFP 14. Fluorescence microscopy revealed strong native eGFP expression in capillaries and small cardiac vessels at both developmental stages (Figure S1A-C). After enzyme digestion of the hearts, cardiac endothelial cells (CECs) were isolated by MACS using CD31 antibodies coupled to magnetic microbeads and plated on glass coverslips. Staining with Hoechst and cell counting demonstrated that one day after plating (d1) there was a much higher number of Hoechst^+^ eGFP^+^ embryonic CECs attached compared to adult CECs (Figure S1D-F). We also performed immunostainings with markers for cell cycle activity (Ki-67) and apoptosis (TUNEL) to compare embryonic and adult CECs. At d7 we found a higher fraction of Ki-67^+^ embryonic CECs (22.5±2.9% (embryonic CECs) vs 14.2±1.7% (adult CECs)) (Figure S1G-I) and a much lower fraction of TUNEL^+^ embryonic CECs (3.8±0.8%, n = 5 (embyronic CECs) vs 16.4±3.0%, n = 5 (adult CECs)) (Figure S1J-L). This indicates an enhanced regenerative potential of embryonic CECs compared with adult CECs. Thus, in the following experiments we concentrated on embryonic CECs.

**Figure 1:**
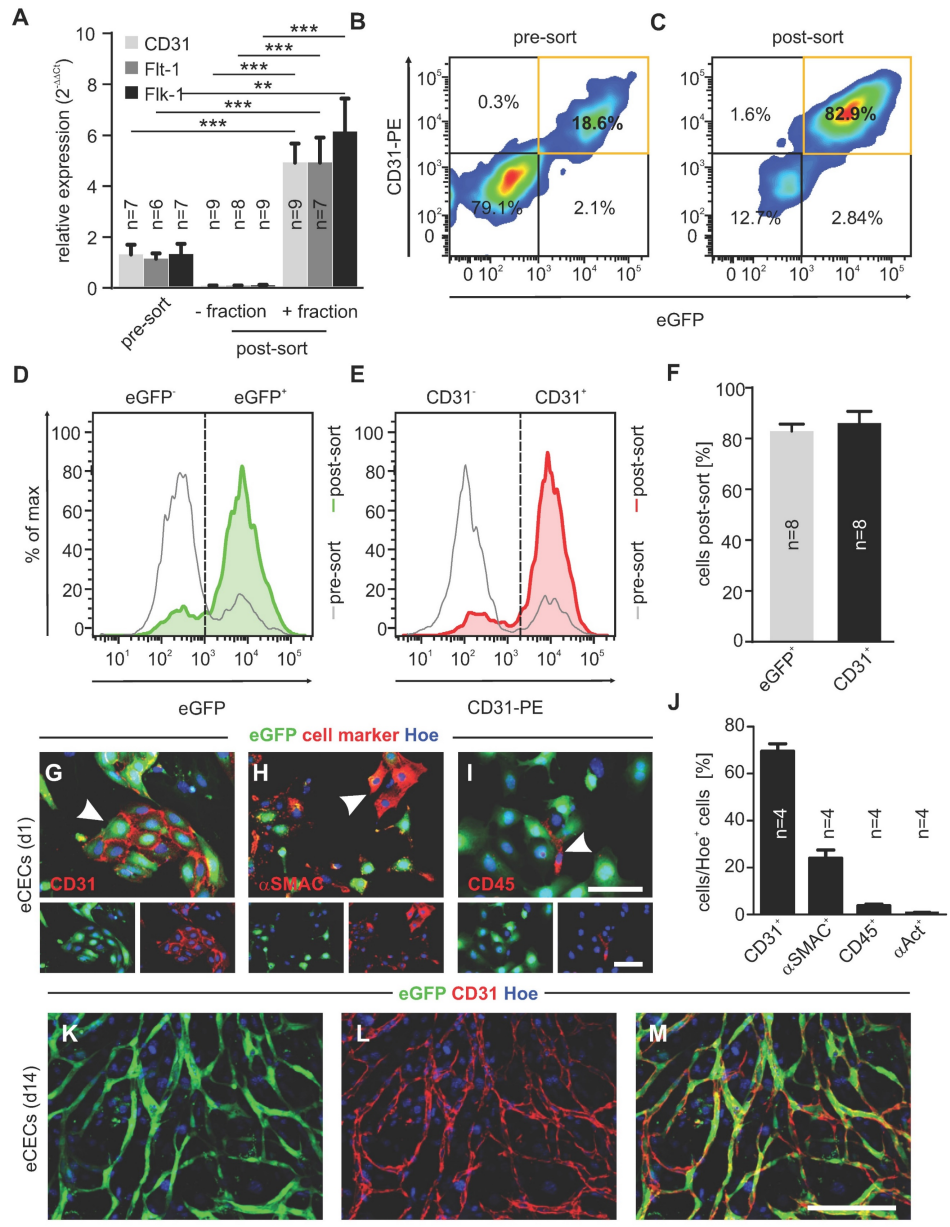
** Embryonic CECs can be highly purified by MACS and display strong angiogenic potential *in vitro*. A)** Quantitative analysis of mRNA expression of EC-specific genes pre- and post-sorting (+: positive fraction, -: negative fraction). **B,C)** Density plots of flow cytometry analysis of CECs before (pre-sort, **B**) and after (post-sort, **C**) MAC sorting. **D,E)** Histograms of eGFP^+^ cells **(D)** and CD31^+^ cells **(E)** pre- and post-sorting. **F)** Quantitative analysis of the amount of eGFP^+^ and CD31^+^ cells after sorting. **G-I)** Immunofluorescence staining of CD31 (red, **G**), asmac (red, **H**) and CD45 (red, **I**) in purified CECs, displaying native eGFP (green) 1d after sorting. **J)** Quantitative analysis of the amount of CD31^+^, asmac^+^, CD45^+^ or a-actinin^+^ cells 1d after sorting. **K-L)** Fluorescence pictures of vascular network formation by CECs on d14, red: CD31, green: native eGFP, blue: Hoechst (Hoe). Bars: 100 µm (G-I, insets, M), **p < 0.01, ***p < 0.001.

To determine the purity of the cells after MACS, we first performed bulk RNA-seq experiments. Principle component analysis revealed very good clustering of embryonic CECs (positive fraction of embryonic hearts), embryonic cardiac cells (CCs, negative fraction of embryonic hearts) and adult CECs (positive fraction of adult hearts) (Figure S2A). A heatmap of genes related to endothelial cell (EC) differentiation 24 showed strong upregulation of endothelial markers in embryonic CECs compared to other cardiac cells (CCs) (Figure S2B). In addition, we performed gene set enrichment analysis (GSEA) and found upregulation of gene sets for endothelium development, EC differentiation and notch signaling in embryonic CECs. Gene sets for cellular respiration, muscle cell development and collagen fibril organization were downregulated in embryonic CECs compared to embryonic CCs, indicating an efficient purification of embryonic CECs by MACS from cardiac cell homogenates (Figure S2C). To corroborate the upregulation of endothelial genes shown by RNA-seq analysis we next performed qPCR. Also here strongly increased expression of the endothelial-specific markers CD31, Flt-1 and Flk-1 (KDR) was found in the positive fraction of embryonic CECs (+ fraction, post-sort) when compared to the negative fraction of cardiac cells (- fraction, post-sort) and to cell homogenates before MACS (pre-sort) (Figure 1A). In order to specify the intramyocardial origin of injected cells as arterial and venous or endocardial and epicardial, we again analyzed our RNA-seq data. As markers for the different cell populations we used Ephrin B2 (Efnb2) for arterial ECs, Eph receptor B4 (Ephb4) for venous ECs 25, Endomucin (Emcn) for endocardial ECs 26 and Apelin (Apln) for subepicardial ECs 27. We found that the majority of CECs directly after isolation are Emcn^+^ endocardial cells (Figure S1D). This was confirmed by qPCR analysis of plated cells on d7 after isolation (Figure S1E).

In addition, we analyzed endothelial marker expression of isolated cells on the protein level. As shown by flow cytometry analysis directly after MACS (post-sort) a strong purification of embryonic CECs reaching about 80% of CD31^+^eGFP^+^ cells was obtained (Figure 1B-F). To better characterize and quantify the cell types of the positive fraction, cells were plated after MACS, followed by immunostainings with markers against ECs (CD31, red, Figure 1G), smooth muscle cells (SMCs)/fibroblasts (asmac, red, Figure 1H), leukocytes (CD45, red, Figure 1I) and cardiomyocytes (α-actinin, Figure 1J) at d1. Cell counting demonstrated that also after cell attachment the vast majority of cells was ECs (69.5±2.7%, n = 4), followed by SMCs/fibroblasts (24.0±3.0%, n = 4), very low numbers of leukocytes (3.7±0.6%, n = 4) and cardiomyocytes (0.9±0.3%, n = 4) (Figure 1J). When plated on Matrigel, eGFP^+^ networks (green) developed over time (Figure 1K,M), and their endothelial identity was proven by CD31 staining (red) (Figure 1L,M). Thus, the purification of embryonic CECs by MACS results in viable ECs with high angiogenic potential.

### Magnet-guided positioning of microbead-labeled CECs *in vitro*

**Figure 2:**
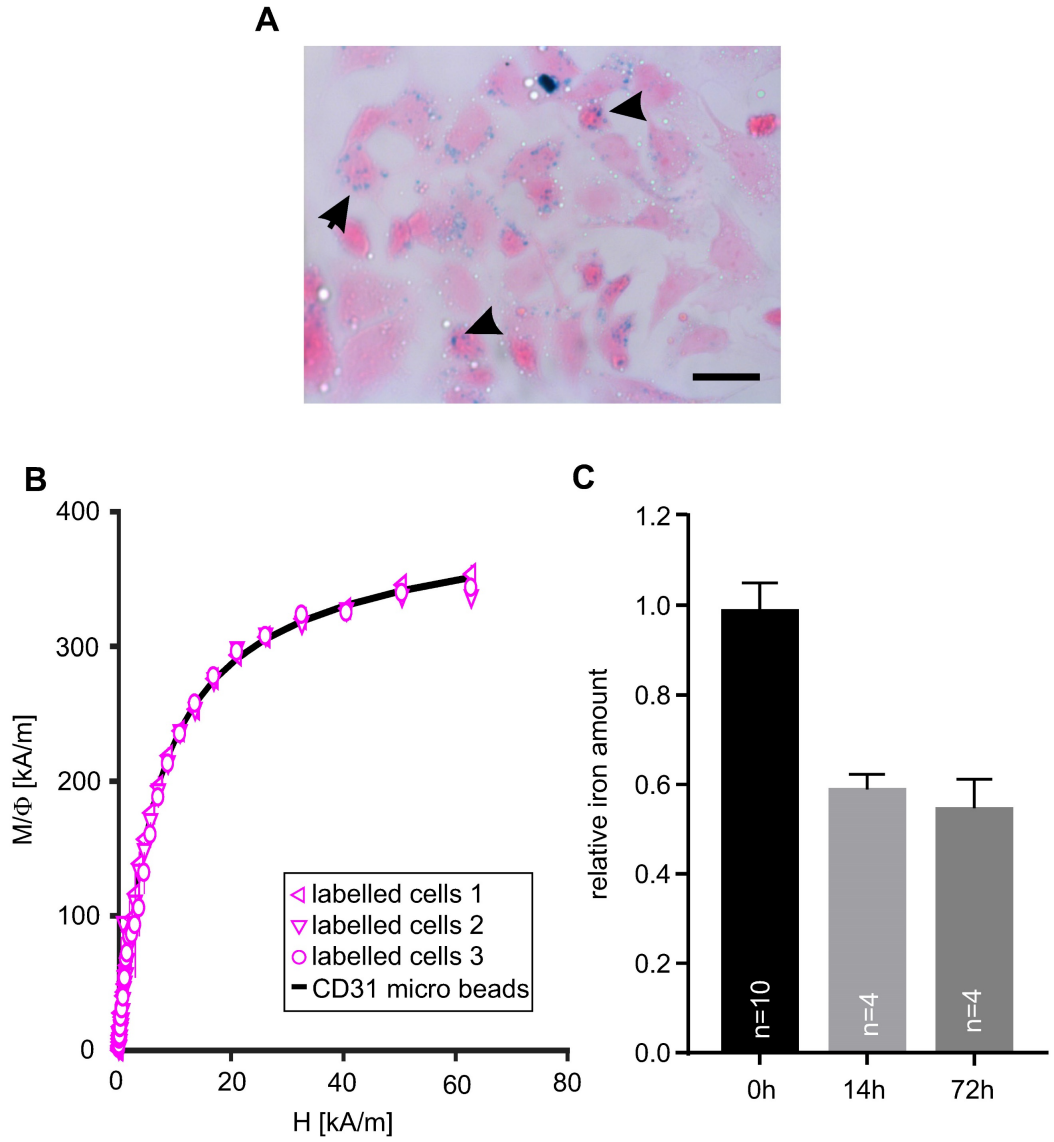
** MACsorted embryonic CECs are labeled with magnetic beads. A)** Prussian blue staining of CECs 6 h after sorting, arrowheads indicate accumulation of microbeads. **B)** Magnetization M(H) curves of magnetic microbeads coupled to anti-CD31 antibodies only and scaled data of MACsorted bead-labeled CECs. Φ: volume fraction of magnetic material, Bar: 50 µm (A). **C)** Quantitative analysis of the relative iron amount of CECs after MACS over time. Bar: 50 µm (A)

Next, we assessed the magnetization of embryonic CECs after MACS-based isolation. Prussian blue staining was applied to visualize MACS microbeads on the cells (Figure 2A, arrows). Then, we quantified the iron amount per cell using magnetic particle spectroscopy (MPS) and found 0.3±0.03 pg Fe/cell (n = 5) in microbead-labeled CECs (Table 1) while no signal was found in control cells. With a microbead core diameter of 30±20 nm 28 this results in about 6,700 beads/cell (Table 1). In another experiment M(H) curves were determined in a static magnetic field and microbead-labeled cells displayed the same shape as control CD31 microbeads, which proves that the static magnetic behavior of microbead-labeled cells is equal to the microbeads alone (Figure 2B). The magnetic moment of the cells amounted to 32.5±3.5 fAm^2^, n = 5 (Table 1). In order to investigate the fate of MACS microbeads bound to CECs *in vitro* we isolated CECs and quantified the iron amount either directly after sorting (0h) and and after plating and cultivation of CECs at 37°C for 14h or 72h. We found a rapid decline of the iron amount/MACS microbeads to about 60 % within the first 14 h but then it remained stable for at least up to 72 h (Figure 2C). This decrease of MACS microbeads can be explained by internalization and subsequent exocytosis or degradation via lysosomes as reported before 29-31.

We also wondered whether the magnetic microbeads attached to CECs after MACS could be exploited for magnet-guided local cell positioning. To test this novel approach, we have designed an experimental setup in which single cell suspensions of microbead-labeled eGFP^+^ CECs were cultured on a shaker with a magnetic tip positioned below the center of the dish (Figure S3A,B). In control experiments no magnet was applied. When a magnet was applied quantification of eGFP^+^ CECs demonstrated that at d1 the magnetic force (+ magnet) had concentrated the cells in the center of the dish (48.9±0.6%, n = 4 within a radius of 1 mm (combined values of areas at 0.5 mm + 1 mm distance from center), Figure S3C,E), whereas much lower densities of eGFP^+^ cells were found in the periphery (Figure S3E). In control experiments (- magnet) eGFP^+^ cells were found to be homogenously distributed throughout the dish with only 21.1±1.7%, n = 4 of the eGFP^+^ cells within a radius of 1 mm (Figure S3D,E). Accordingly, at d10 eGFP^+^CD31^+^ vascular networks were predominantly found in the center (Figure S3F,G) but not the periphery (Figure S3F,H) of the dish when a magnet had been applied. In controls without a magnet eGFP^+^ vascular networks were found at different localizations throughout the dish (Figure S3I-K). Quantification revealed that application of the magnet concentrated eGFP^+^ branching points (63.1±3.7%, n = 4) and capillary loops (65.9±4.8%, n = 4) within a radius of 1 mm around the center of the dish. In no magnet controls only 14.3±2.0%, n = 4 of the branching points and 5.5±2.2%, n = 4 of the capillary loops could be detected in this area (Figure S3L,M). Thus, magnetic labeling of CECs by MACS microbeads can have a dual role: it enables prominent EC purification as well as the local positioning of the cells by magnetic fields. Next, we exploited these cells for site-directed vascular network formation in an experimental approach of vascular regeneration.

### Magnet-guided transplantation of CECs into myocardial infarction

**Figure 3:**
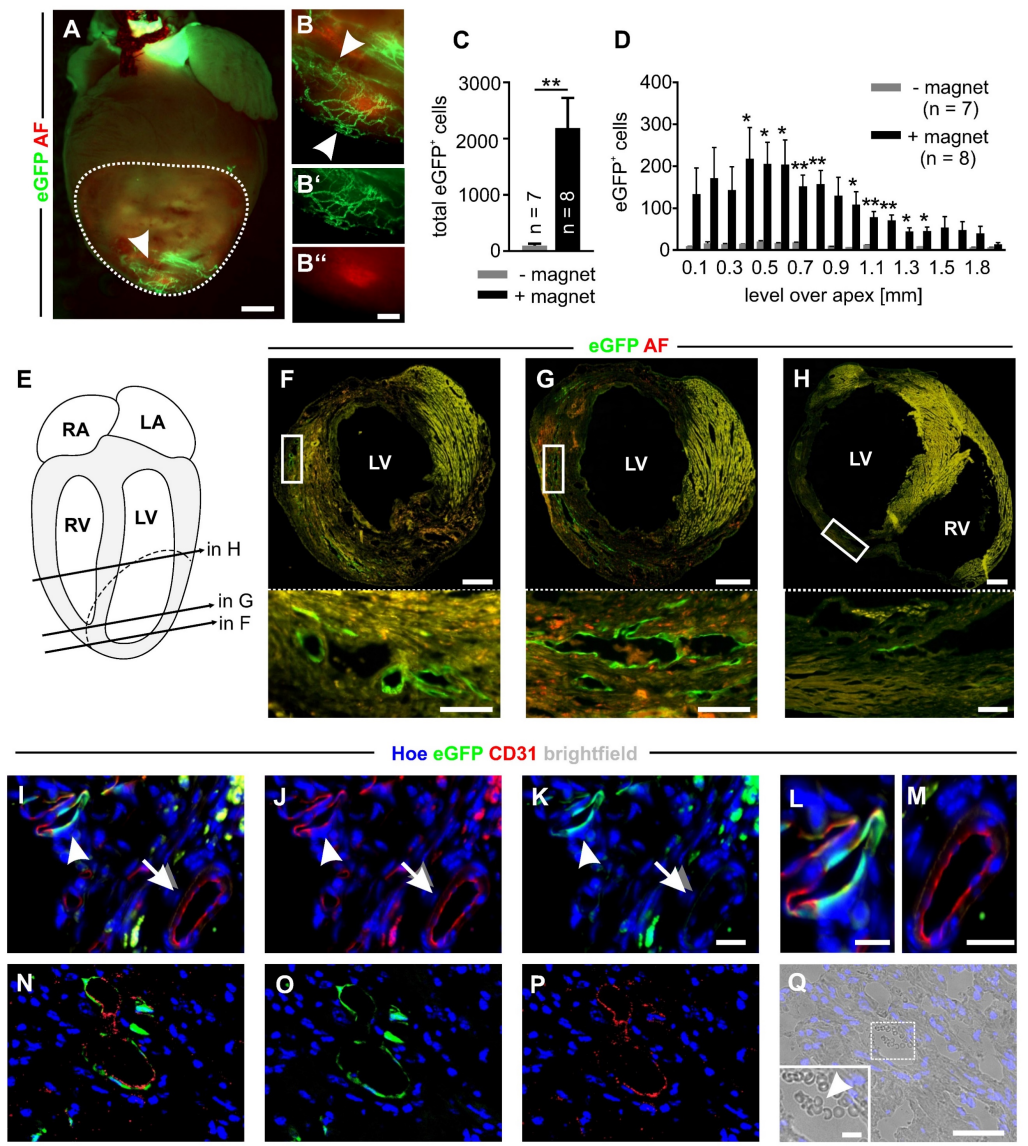
** Magnet application strongly increases embryonic CEC engraftment in myocardial infarction and results in perfused eGFP^+^ vessels. A)** Fluorescence picture of a mouse heart on d14 after CEC injection with magnet application. The dotted line labels the infarct area, the arrowhead points towards eGFP^+^ networks. **B)** Close-up of the region indicated by the arrowhead in A), arrowheads label vascular networks, B`) green channel of the vascular network region, B``) red channel of the vascular network region. **C)** Quantitative analysis of the total number of eGFP^+^ cells in the infarct area after CEC injection with (+) or without (-) magnet application. **D)** Quantitative analysis of the distribution of eGFP^+^ cells in the infarct area after injection + or - magnet application. **E)** Schematic diagram of the heart indicating the position of the sections shown in F-H), RA = right atrium, LA = left atrium, RV = right ventricle, LV = left ventricle, dashed line = infarct area. **F-H)** Fluorescence pictures of heart sections treated with CECs + magnet application, sections derived from the positions indicated in E), top: overview of the section, bottom: close up of the regions indicated by the boxes in the pictures above, green = native eGFP, red = autofluorescence (AF). **I-K)** Immunostaining of a heart section of the +magnet group displaying an eGFP^+^CD31^+^ vessel (arrow head) next to an eGFP^-^CD31^+^ vessel (arrow). **L,M)** Close ups of the vessels indicated by the arrow head **(L)** and arrow **(M)** in **I-K. N-P)** Immunostaining of a heart section of the + magnet group with an eGFP^+^CD31^+^ vessel. **Q)** Brightfield picture of the vessel shown in **N-P)**, inset: close up of the white box, arrow head indicates erythrocytes in the lumen area of the eGFP^+^CD31^+^ vessel, green = native eGFP **(I,K,L,M,N,O)**; red = CD31 **(I,J, L, M, N, P)**, blue = Hoechst (Hoe) **(I-Q)**. Bars: 1000 µm **(A)**, 500 µm **(F-H, top)**, 300 µm (B``), 100 µm **(F-H, bottom)**, 50 µm **(Q)**, 20 µm **(K, M)**, 10 µm **(L, inset Q)**, *p < 0.05, **p < 0.01.

To test magnet-guided transplantation of microbead-labeled cells *in vivo*, we generated myocardial cryoinfarctions in mice and injected 200,000 MACsorted embryonic CECs into the lesion in the anterolateral left ventricular wall. A small 1.3 T bar magnet was positioned at a distance of approximately 5 mm from the injection site for 10 min 6. To illustrate the strength of the bar magnet we calculated the gradient of the magnetic flux density and found that the magnet generated a strong and homogenous gradient field that declines with distance (Figure S4A). In controls, no magnet was used. At d14 after infarction hearts were isolated and cell engraftment was analyzed (Figure S4B-D). Fluorescence pictures of the hearts demonstrated eGFP^+^ vascular networks in the infarct area only when a magnet was applied (+ magnet) (Figure 3A,B). To quantify the number of engrafted CECs, eGFP^+^ cells were counted in cryosections of the infarct area. We found 2184±541 (n = 8) eGFP^+^ cells (ranging from 470 to 4177 cells) when a magnet was applied (+ magnet), which was a 23-fold increase of engrafted cells compared to controls without a magnet (- magnet) (94±29 eGFP^+^ cells, n = 7) (ranging from 13 to 273 eGFP^+^ cells) (Figure 3C). In addition, the analysis revealed that there was a position-dependent enrichment of the cells in the + magnet group (Figure 3D). In transversal sections of the hearts, we detected eGFP^+^ vascular-like structures forming lumina at different levels over the apex (Figure 3E-H). Immunohistochemistry demonstrated an overlap of native eGFP with CD31 staining, confirming the endothelial nature of eGFP^+^ cells in the infarct area (Figure 3I-K, arrowheads, L). Besides these eGFP^+^CD31^+^ vascular structures that appeared to be newly formed by eGFP^+^ CECs, also eGFP^-^CD31^+^ vessels were detected that represent the pre-existing vasculature (Figure 3I-K arrows, M). In addition, in some lumina of eGFP^+^CD31^+^ vessels (Figure 3N-P) erythrocytes could be found, indicating that these vessels were connected to the pre-existing vasculature (Figure 3Q). In order to determine the fate of CECs and MACS microbeads after magnet-assisted transplantation of the cells we quantified nanoparticular iron by MPS in the CECs before injection, and in different tissues (the infarct area, the remainder of the heart, the liver and the spleen) directly (15 min) and 48h after injection. We could detect similar amounts of nanoparticular iron in the infarct area of 3 out of 7 hearts (1.5±0.3 ng, n = 3, 2 hearts after 15 min and 1 heart after 48h) and found no signal in all the other tissue samples. This iron amount corresponds to about 7900 CECs and indicates that even though a magnet was used the majority of the cells is lost during injection. The lack of signals in all the other tissue samples can be most likely explained by iron amounts below the detection limit of the MPS method. Thus, there is no indication for a relevant iron accumulation in the heart or other organs.

### Effect of magnet-guided CEC injection on infarct size and heart function

**Figure 4:**
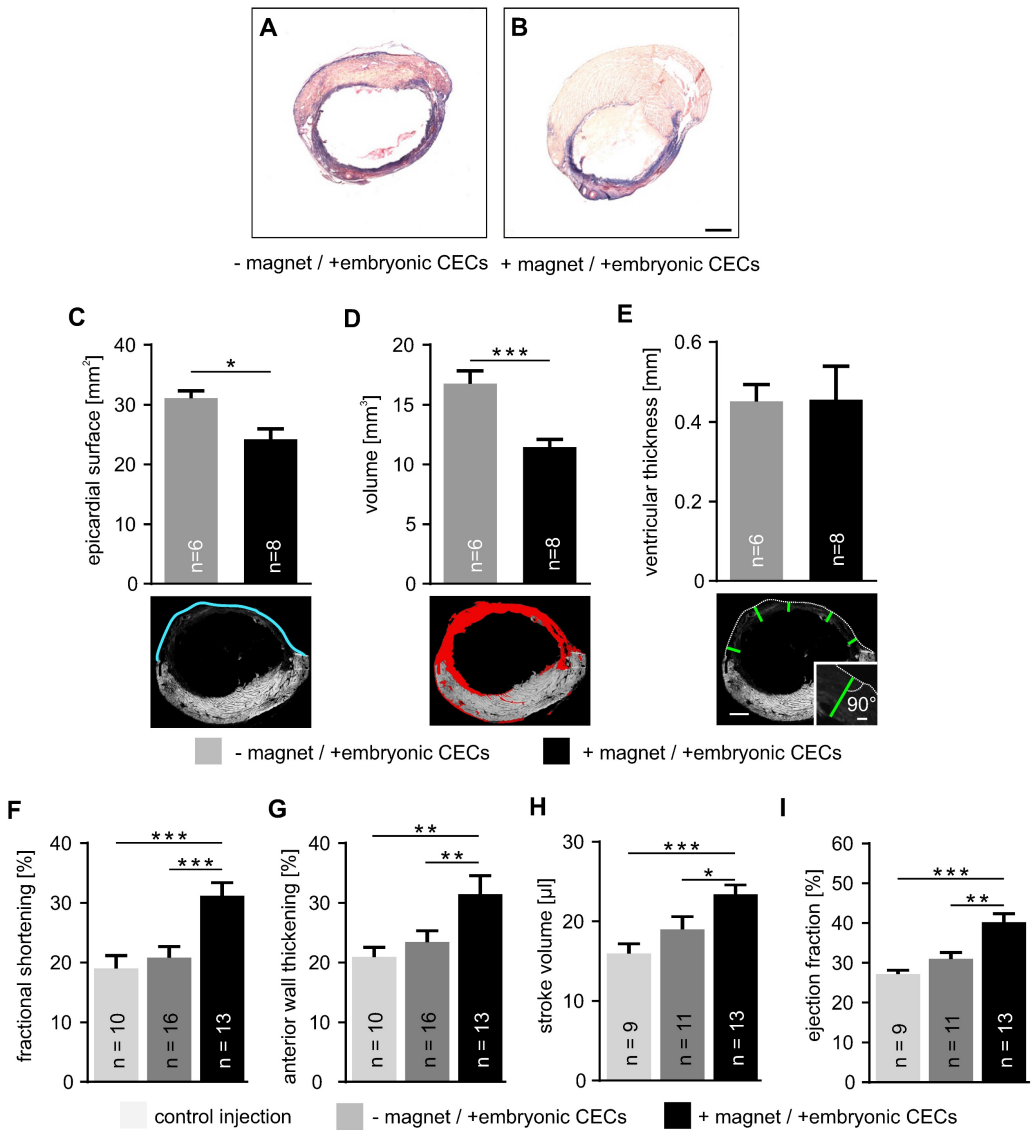
** Embryonic CEC transplantation into myocardial cryoinfarction reduces infarct size and improves cardiac function. A,B)** Masson trichrome staining of representative transversal sections taken from cryoinjured hearts + or - magnet application after cell transplantation. **C-E)** Analysis of infarct size, top: quantitative analysis of epicardial surface **(C)**, volume **(D)** and ventricular thickness **(E)**, bottom: sample pictures illustrating the measurements, blue = epicardial surface length, red = infarct area, green = thickness of vascular wall at 5 different localizations, same heart as shown in Figure 4A. **F-I)** Quantitative analysis of fractional shortening **(F)**, anterior wall thickening **(G)**, stroke volume **(H)** and ejection fraction **(I)**. Bars: 1 mm (B, E, bottom), 100 µm (E, inset). *p < 0.05, **p < 0.01, ***p < 0.001.

Next, we analyzed infarct size after CEC injection into cryoinfarcted hearts by morphometric analyses. We have chosen the experimental model of myocardial cryoinfarction because it is characterized by very reproducible lesion sizes. This enables the reliable quantification of tissue injury and heart function. Hearts with CEC injection - and + magnet application were compared (Figure 4A,B). Our analysis demonstrates that the epicardial surface area of the infarct in CEC-injected hearts was reduced when a magnet was applied (+ magnet: 24.2±1.7 mm^2^, n = 8 vs - magnet: 31.1±1.3 mm^2^, n = 6, p < 0.05) (Figure 4C). Similarly, infarct volume was smaller when CECs were injected in the presence of a magnetic field (+ magnet:11.4±0.7 mm^3^, n = 8 vs - magnet: 16.8±1.1 mm^3^, n = 6, p < 0.001) (Figure 4D). In contrast, there was no change of the thickness of the ventricular wall of the lesion (Figure 4E). This indicates that CEC engraftment promotes tissue repair and remodeling reducing infarct size but unlike other cell types with larger size/volume (cardiomyocytes, skeletal myoblasts) 32-34 CECs cannot restore ventricular wall thickness.

These morphometric analyses were also correlated with heart function determined by echocardiography and LV pressure-volume (PV) catheter measurements. Analysis by motion (M) mode echocardiography revealed a prominent increase of fractional shortening in the + magnet group compared to controls or CEC injections in the - magnet group (+ magnet: 31.7±1.8%, n = 13 vs - magnet: 20.8±1.9%, n = 16, p < 0.001) (Figure 4F). Similar results were found for anterior wall thickening (+magnet: 32.3±2.4%, n = 13 vs -magnet: 23.5±1.9%, n = 16, p < 0.01) that reflects regional heart function 17 (Figure 4G). Also PV measurements with a 1.4F Millar catheter demonstrated that CEC injections + magnet improved stroke volume (+ magnet: 23.4±1.2 µl, n = 13 vs - magnet: 19.0±0.7 µl, n = 11, p < 0.05) (Figure 4H). Furthermore, the ejection fraction increased when a magnet was applied during cell injection (+ magnet: 40.2±2.1%, n = 13 vs - magnet: 30.9±1.7%, n = 11, p < 0.01) (Figure 4I). Thus, CEC injection in combination with a magnetic field strongly reduces infarct size and improves heart function.

**Figure 5:**
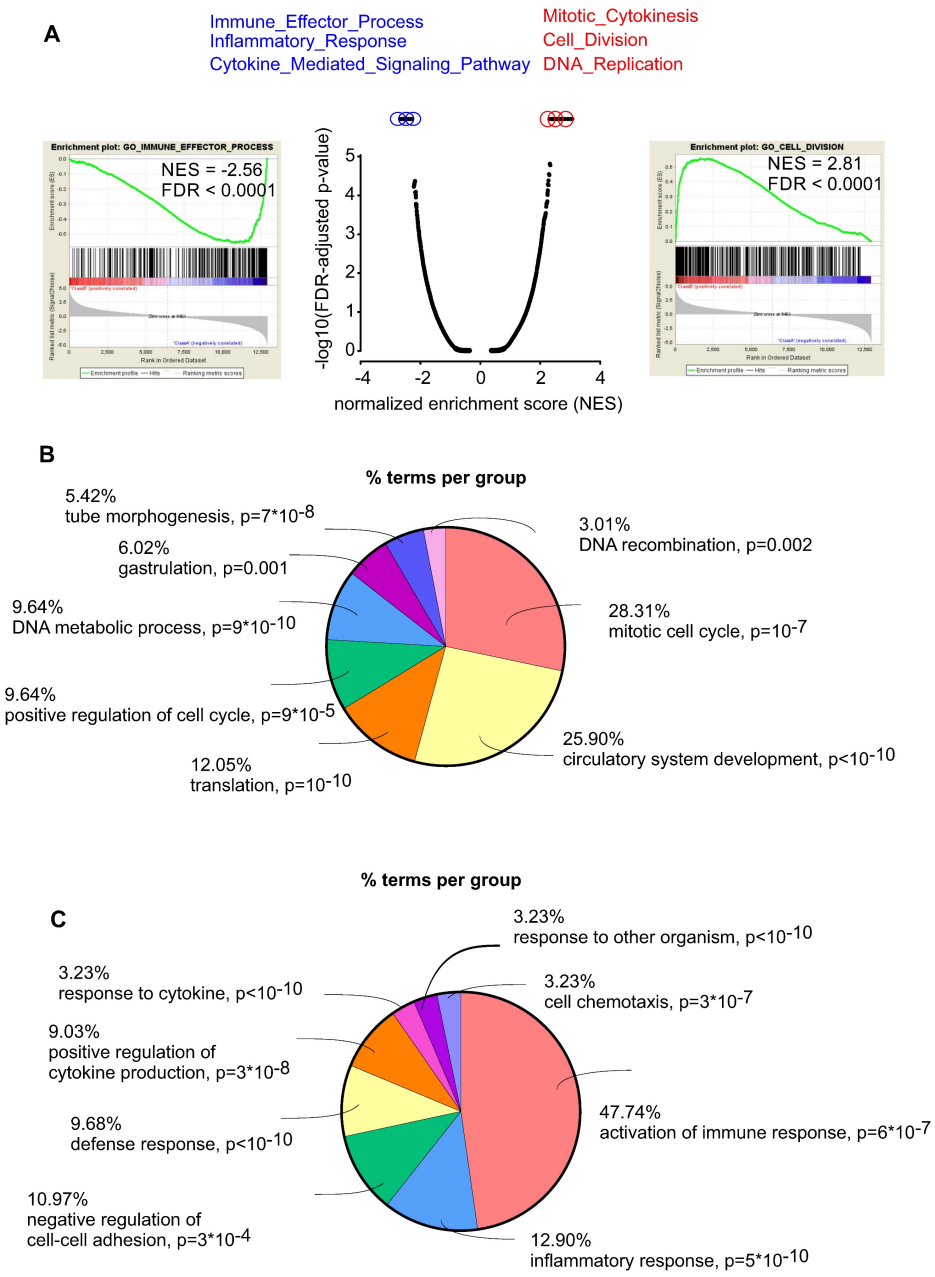
** Embryonic CECs have pro-regenerative and anti-inflammatory properties. A)** Gene set enrichment analysis (GSEA) of upregulated (red) and downregulated (blue) gene sets in embryonic CECs vs adult CECs. **B)** Gene ontology (GO) analysis of the category “biological processes” of the 300 most upregulated genes in embryonic CECs (n = 5) vs adult CECs (n = 6). **C)** Gene ontology (GO) analysis of the category “biological processes” of the 300 most downregulated genes in embryonic CECs vs adult CECs.

To better characterize the mechanism by which embryonic CECs contribute to regeneration of myocardial infarction we then performed RNA-seq analysis to compare embryonic (n = 5) and adult (n = 6) CECs. We found 1854 upregulated and 2930 downregulated genes in embryonic CECs (Figure S5). Gene set enrichment (GSEA) analyses revealed that embryonic CECs displayed a strong upregulation of gene sets for mitotic cytokinesis, cell division and DNA replication, which is in accordance with their high regenerative potential. The most downregulated gene sets in embryonic CECs were related to immune effector processes, inflammatory response and cytokine-mediated signaling (Figure 5A). This suggests that embryonic CECs possess anti-inflammatory properties, that have also been proposed to underlie at least in part the beneficial role of injected mesenchymal stem cells in myocardial infarction 35,36. We also performed gene ontology (GO) analyses with terms related to biological functions. In accordance with our results from GSEA in embryonic CECs, we found enrichment of terms related to mitotic cell cycle and circulatory system development (Figure 5B) as well as downregulation of terms reflecting activation of immune response and inflammatory response (Figure 5C), which confirms the pro-regenerative and anti-inflammatory effects of embryonic CECs.

## Discussion

The regenerative capacity of the heart after injury is very limited. Therefore, numerous cell replacement strategies have been developed. Thereby, different cell types were applied for cell transplantation into myocardial infarction in animal studies 37 but also in clinical trials in humans 38,3. All of these cell types have certain advantages and disadvantages, but a main problem, in particular for human applications, is the limited availability of the cells, considering the extremely low engraftment rates in the heart 39. To enhance cell engraftment various interesting magnetic targeting approaches have been developed 40-42. Another problem is that similar experimental approaches often provided inconsistent results, which may be explained by application of divergent and heterogeneous cell populations 43. To improve the reproducibility of the outcome of cell replacement strategies, more defined cell populations have to be chosen. For that purpose, cell isolation by MACS using specific antibodies is a promising strategy.

Thus, in a proof-of-concept study we have used an unprecedented approach and applied labeling of cells by commercially available MACS microbeads for both, the purification of the CEC population and the enhancement of CEC engraftment into myocardial infarction by application of a magnetic field. We have chosen early CECs because these cells could combine restoration of the vascular network by direct formation of new blood vessels and beneficial paracrine effects on the surrounding tissue. To avoid immunological problems, ethical issues and because of their availability in high numbers induced pluripotent stem cells (iPS) would be an ideal autologous source of early CECs and other cell types of the cardiovascular lineage for future therapeutic cell transplantations 44. Our RNA-seq and qPCR data revealed that MACsorting with bead-labeled antibodies directed against an endothelial surface antigen provides high purity CECs. Residual fibroblasts in the cell preparations may be even beneficial as in an earlier study we could show that purified embryonic stem cell-derived cardiomyocytes displayed a much better engraftment in the injured myocardium when injected together with fibroblasts 32. Previous studies in mice and humans demonstrated that microbeads alone or stem cells isolated by MACS and therefore decorated with microbeads showed no deleterious effects on cardiac regeneration after cardiac injury 29,45. In our experiments we injected 200,000 isolated CECs corresponding to iron amounts below 60 ng and this is much less than those iron amounts that showed toxic effects on the heart before 46-47. While *in vitro* studies demonstrated that microbeads, at least on non-dividing cells, persist on the cell surface for up to two weeks 48, MACsorted stem cells have been reported to lose their beads within 48 h of transplantation into myocardial infarction 29 and most likely enter the individual´s iron metabolism. Also in our *in vitro* study we found a rapid decline of MACS microbeads on CECs during the first hours after isolation and magnet-assisted transplantation of MACsorted CECs resulted in relatively low absolute numbers of cells and microbeads in the infarct area. This suggests that further optimization of the injection procedure is required (stronger magnetic field, longer magnet application etc.). When comparing the surface labeling strategy using MACS microbeads with the internalization method where magnetic particles are taken up by the cells via endocytosis, surface labeling results in much lower iron amounts per cell. This can be recognized in Prussian blue stainings by us and others 9, 40-41. In fact, the iron amount per CEC obtained by the surface labeling strategy in this study was 10 times lower than that of a cardiomyocyte that was loaded with MNPs after internalization in our previous study 6. Still the magnetic moment of the CECs in this study was high enough for local positioning of the cells by a magnetic field *ex vivo* and for a strong enhancement of CEC engraftment in the myocardial infarction *in vivo*. With a 23-fold increase of CEC engraftment in myocardial infarction this procedure was even more efficient than that used for MNP-loaded cardiomyocytes where only a 7-fold increase could be obtained 6. This difference may be explained by the different cell types applied, as small sized CECs show very poor engraftment under control conditions. The lower iron amount per cell using the surface labeling strategy may also be beneficial to retain a proper cell function as the internalization method had been reported to potentially induce adverse effects 49. Yet, depending on their size also some of the beads used for surface labeling may be internalized over time 29,49. To examine both labeling strategies in detail these need to be compared in the same cells type and injury model in future studies. The absence of adverse effects by surface labeling in this study was also demonstrated by engrafted eGFP^+^ CECs forming extensive vascular networks in the heart when a magnet was applied and some of these eGFP^+^ structures contained erythrocytes in their lumina suggesting a connection with the pre-existing coronary vasculature. Alternatively, eGFP^+^ CD31^+^ vessels could have been generated by fusion of eGFP^+^ CECs with resident CECs in vessels. Such a fusion has been reported for bone marrow cells with cardiomyocytes 50 or embryonic stem cells 51 and seems unlikely but cannot be completely excluded. We can show that enhanced CEC engraftment by application of a local magnetic field resulted in an improvement of cardiac function and reduction of infarct size. If these changes are due to increased perfusion by newly formed vessels connected to the pre-existing vasculature and/or paracrine effects of transplanted cells on CECs, cardiomyocytes or resident stem cells is currently unclear and both options were also discussed for transplantations of other cell types 52. Irrespective of the underlying mechanism, we observed an improvement of cardiac function only when the number of engrafted cells was increased by magnet application. Future studies will have to reveal for how long CECs and newly formed vessels will persist in the myocardium. Our previous study with transplantation of MNP-loaded cardiomyocytes demonstrated that application of a magnetic field strongly enhances long-term engraftment of the cells for up to 8 weeks 6. In case that paracrine effects of CECs on resident cells are mainly responsible for the improvement of heart function, a prolonged but transient engraftment could be sufficient for sustained functional repair. There are some limitations of our study that will have to be addressed in future work. We used the cryoinjury model for experimental myocardial infarction because it produces a highly reproducible lesion size that can be easily quantified to examine the effect of therapeutic cell transplantation. However, cryoinjury generates a necrotic wound which does not recapitulate the pathophysiology of myocardial infarction in humans. Therefore, an experimental model generating ischemia (e.g. left anterior descending artery (LAD) ligation) would be more representative for the human situation. Our study shows beneficial effects of magnet-assisted cell transplantation of 200,000 cells after MACS-based isolation in mice. For large animal models or even humans many more would be required. These cell numbers could be difficult to obtain via current MACS procedures. Thus, further technical advances are needed for translation.

## Conclusions

Taken together, the combined use of magnetic microbeads for isolation of cells by MACS and for the improvement of *in vivo* cell engraftment by application of a magnetic field is a powerful novel approach to optimize cell transplantation strategies in the heart.

## Supplementary Material

Supplementary figures.

## Figures and Tables

**Table 1:** Characteristics of microbead-labeled embryonic CECs.

iron load/cell [pg]	0.29±0.03	n=5
beads/cell	6715.6±723.3	n=5
magnetic moment/cell [fAm^2^]	32.5±3.5	n=5
