# Supplementary material for: Combined use of magnetic microbeads for endothelial cell isolation and enhanced cell engraftment in myocardial repair
Source: Theranostics. 2023 Feb 5;13(3):1150–64. doi: 10.7150/thno.75871 (PMC9925315; doi:10.7150/thno.75871)
Supplement: Supplementary file 1 — Supplementary figures. [file thnov13p1150s1.pdf]

Supplementary Material

Combined use of magnetic microbeads for endothelial cell isolation and enhanced cell engraftment in myocardial repair

Christian Biederbick[1#], Jan C. Heinemann[1,2#], Sarah Rieck[1], Florian Winkler[1], Annika Ottersbach[1,2], Miriam Schiffer[2], Georg D. Duerr[2,3], Dietmar Eberbeck[4], Michael Hesse[1], Wilhelm Röll[2], *Daniela Wenzel[5,1*]

[1]Institute of Physiology I, Life&Brain Center, Medical Faculty, University of Bonn, Bonn, Germany

[2]Department of Cardiac Surgery, Medical Faculty, University of Bonn, Bonn, Germany

[3]Department of Cardiovascular Surgery, University Medical Center Mainz, Johannes Gutenberg-University, Mainz, Germany

[4]Physikalisch-Technische Bundesanstalt (PTB), Berlin, Germany

[5]Institute of Physiology, Department of Systems Physiology, Medical Faculty, Ruhr University of Bochum, Bochum, Germany

#contributed equally

*Corresponding author:
Daniela Wenzel, MD
Institute of Physiology, Department of Systems Physiology
Medical Faculty, University of Bochum
Universitätsstr. 150
44801 Bochum
Germany
Tel: 0049/234/32-29100
Email: daniela.wenzel@rub.de

Supplementary Figures

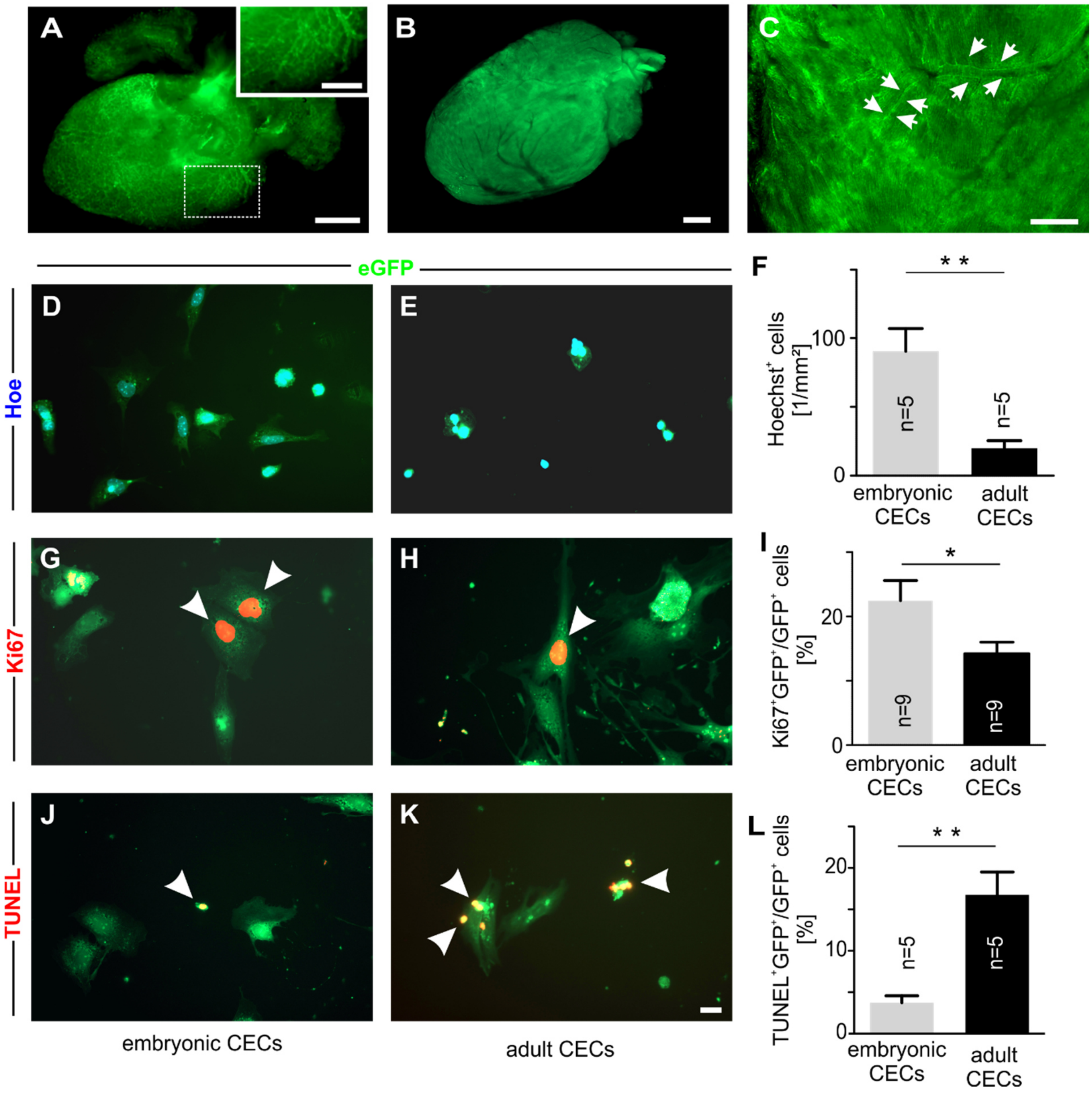

Figure S1. Embryonic CECs show a higher regenerative potential than adult CECs in vitro. A) Fluorescence image of embryonic heart derived from the flt1/eGFP mouse line, inset: eGFP$^+$ CEC network, region of the dashed box shown in A. **B)** Fluorescence image of adult heart derived from the flt1/eGFP mouse line. **C)** Close up of adult heart with eGFP$^+$ capillaries larger vessels (arrows). **D,E)** Fluorescence pictures of hoechst$^+$ embryonic **(D)** and adult CECs **(E)** at d1. **F)** Quantitative analysis of hoechst$^+$ embryonic and adult CECs at d1. **G,H)** Fluorescence pictures of Ki-67$^+$ (arrow heads) embryonic **(G)** and adult CECs **(H)** at d7. **I)**

Quantitative analysis of Ki-67$^+$ embryonic and adult CECs at d7. **J,K)** Fluorescence pictures of TUNEL$^+$ (arrow heads) embryonic **(J)** and adult CECs **(K)** at d7. **L)** Quantitative analysis of TUNEL$^+$ embryonic and adult CECs at d7. Bars: 20 μm (K), 500 μm (A,C), 250 μm (Inset A), 1000 μm (B), *$p < 0.05$, **$p < 0.01$.

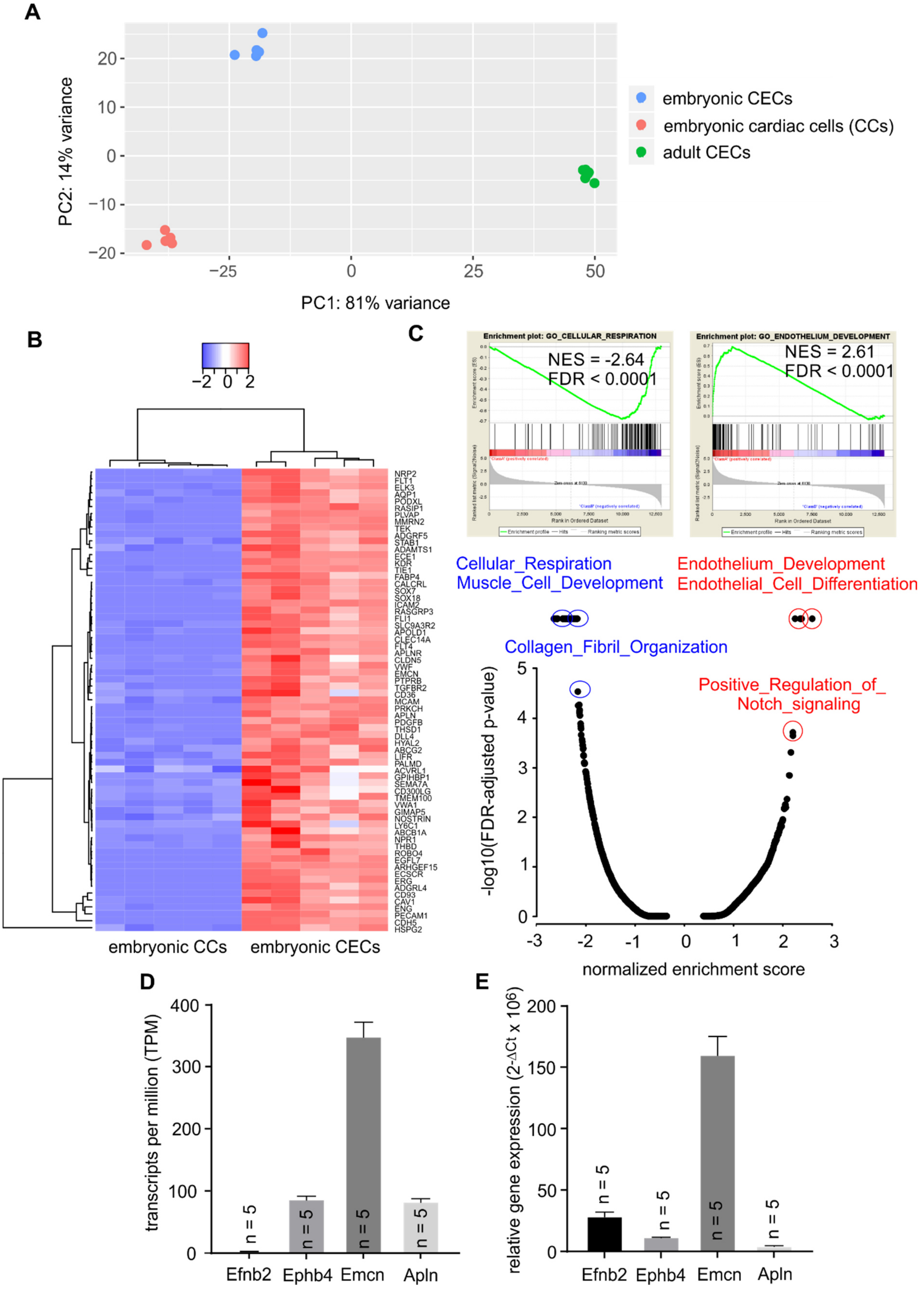

Figure S2. RNA-seq analysis confirms high purity of MACsorted embryonic CECs. A) Principal component analysis of MACsorted embryonic CECs (positive fraction, blue, n=5),

embryonic cardiac cells (CCs, negative fraction, red, n = 5) and adult CECs (positive fraction, green, n = 6). **B)** Heatmap of endothelial gene expression of embryonic CECs compared to embryonic CCs. **C)** Differential gene expression pattern of embryonic CECs (red) and embryonic CCs (blue) shown by gene set enrichment analysis. **D,E)** Gene expression pattern of endothelial markers characterizing injected CECs either generated by RNAseq directly after isolation (**D**) or generated by qPCR on d7 after plating (**E**).

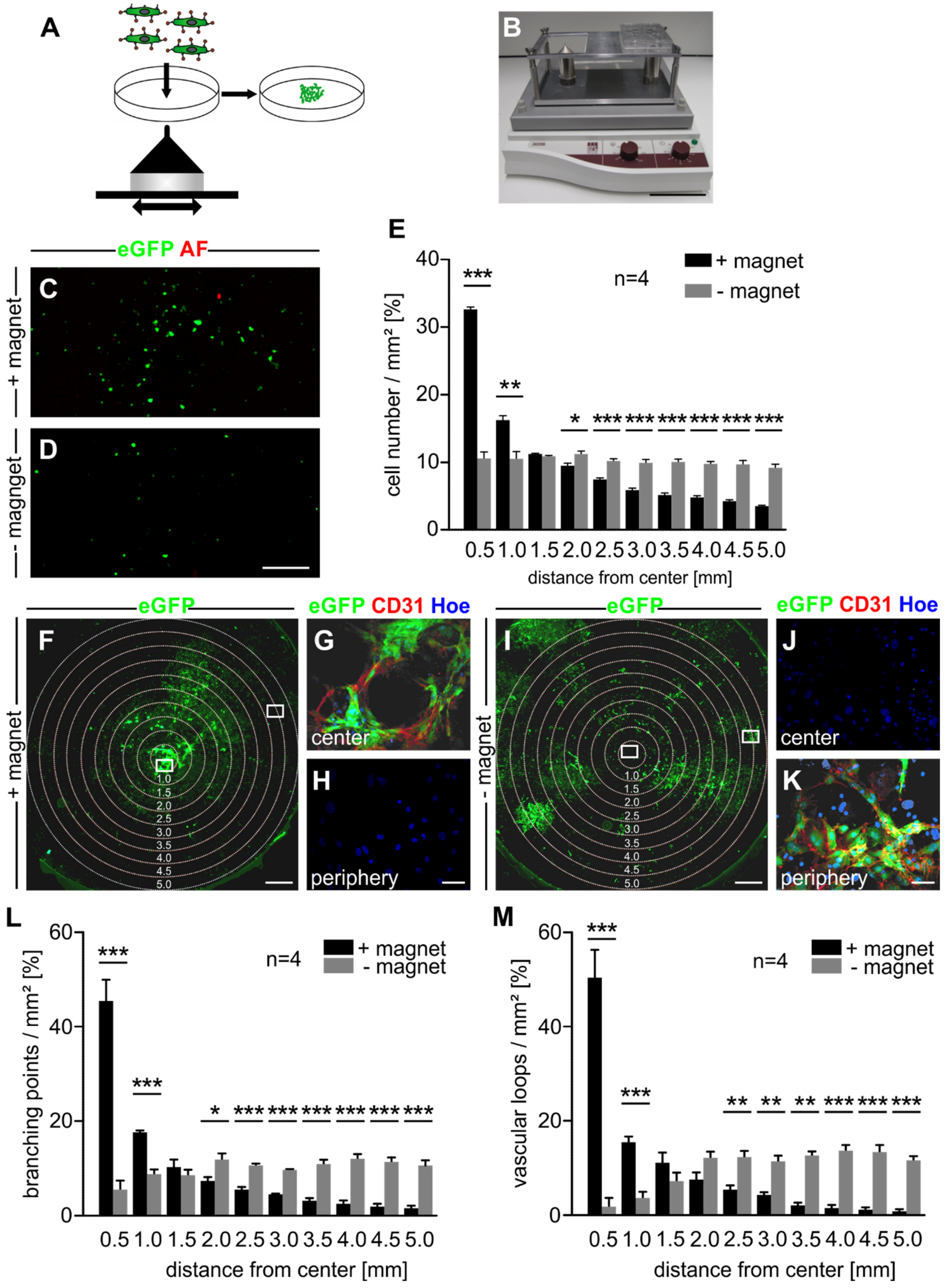

Figure S3. MACsorted CECs can be locally positioned by a magnetic field in vitro. A,B)

Schematic view **(A)** and picture **(B)** of the experimental set-up. CECs were isolated, purified

via MACS and transferred to a shaking culture dish with a magnetic tip underneath. **C,D)** Fluorescence images of eGFP$^+$ cell distribution at d1 when a magnetic field was applied (+ magnet) **(C)** or when no magnetic field was applied (- magnet) **(D)**. **E)** Quantitative analysis of eGFP$^+$ cell number depending on the distance from center of the magnetic tip on d1. **F-H)** Cell distribution at d10 when a magnet was applied during plating (+ magnet), overview **(F)** and magnified regions of the center **(G, white box in F)** and periphery **(H, white rectangle in F)**. **I-K)** Cell distribution at d 10 when no magnet was applied during plating (- magnet), overview **(I)** and magnified regions of the center **(J, white box in I)** and periphery **(K, white rectangle in I)**. **L,M)** Quantitative analysis of branching points **(L)** and capillary loops **(M)** at d10. The numbers within the white circles in **F)** and **I)** indicate the distance from the center where branching points and loops were quantified. Green = native eGFP, red = autofluorescence (AF) in C,D, red = CD31 in G,K, blue= hoechst (Hoe); bars = 10 cm (B), 200 µm (D), 50 µm (H, K), 1000 µm (F,I), *$p < 0.05$, **$p < 0.01$, ***$p < 0.001$.

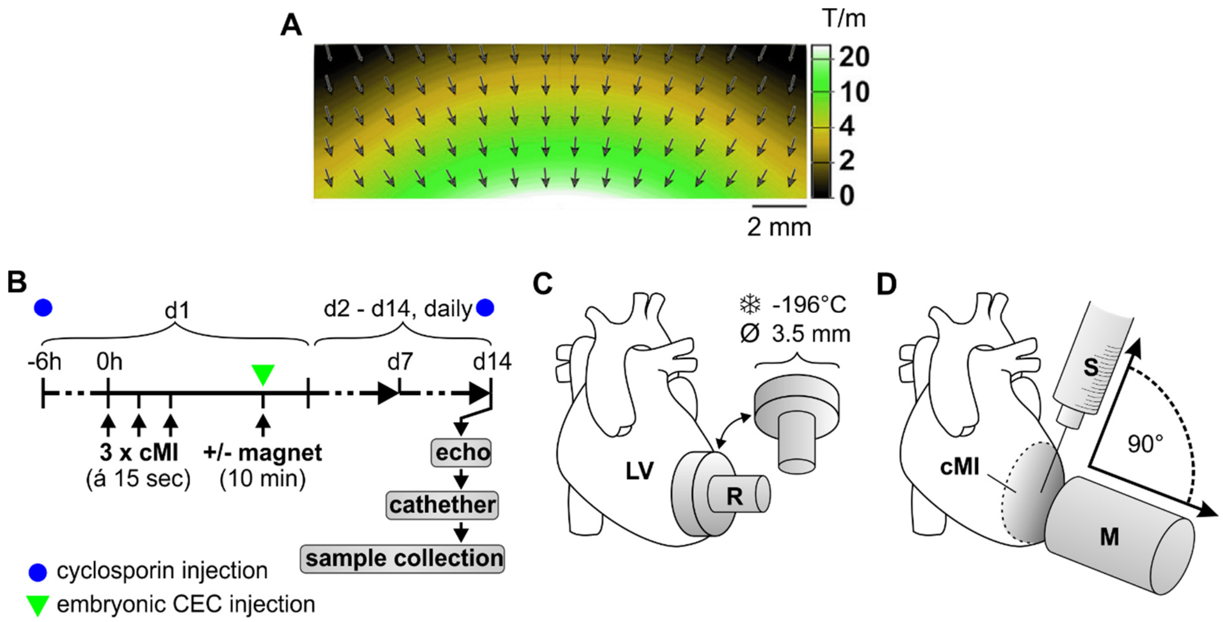

Figure S4. Schematic diagram of procedures for embryonic CEC transplantation experiments. A) Characterization of magnetic field generated by 1.3 T bar magnet: Gradient of magnetic flux density at a distance of 5 mm. **B)** Time schedule of surgical procedure and analysis. An experimental myocardial cryoinfarction (cMI) is induced by application of an ice-cold copper rod on the apex region of a murine heart (3 x 15 sec). Then, CECs are injected and a small magnet (1.3 T) is placed about 5mm above the injection site for 10 min. To prevent cell rejection, cyclosporin is applied daily. On day 14 heart function is determined by echocardiography and left ventricular pressure volume catheter measurements. After that, the hearts are harvested for histology and morphometry. **C)** Schematic diagram illustrating the generation of experimental cryoinfarctions. A copper rod with a diameter of 3.5 mm is cooled in liquid nitrogen (-196°C) and then pressed three times on the apex region of the left ventricular wall (LV). **D)** Schematic diagram illustrating cell injection and magnet application. CECs are injected into the cMI by use of a Hamilton syringe (S) while a small magnet (1.3 T, M) is positioned at a short distance above the injection site.

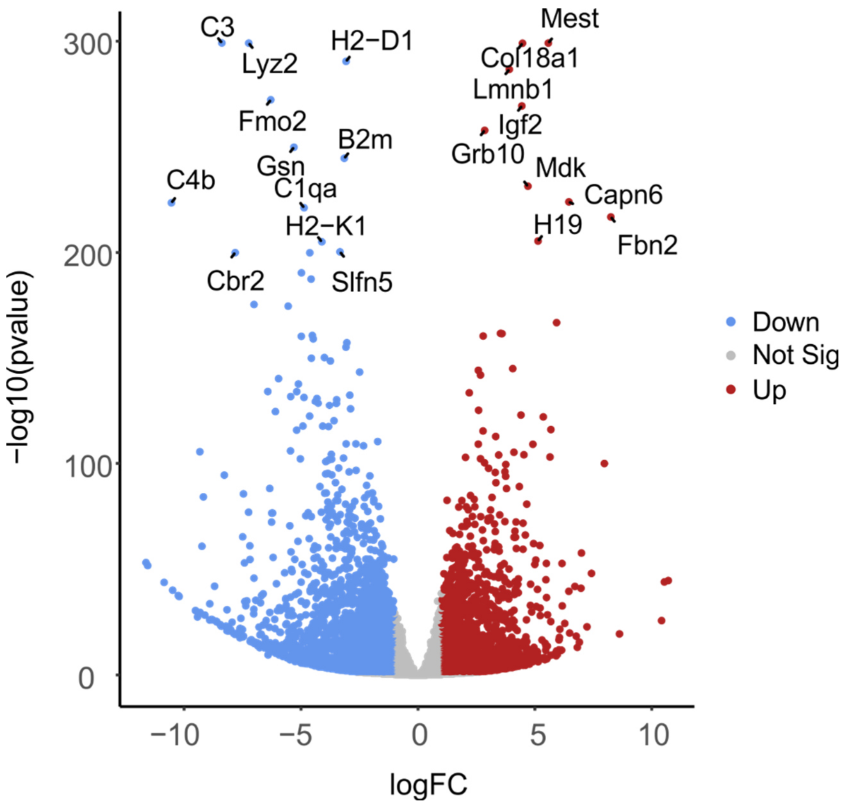

Figure S5. Volcano plot of differentially regulated genes in embryonic vs adult CECs. The volcano plot reveals 1854 upregulated genes (red) and 2930 downregulated genes (blue) in embryonic vs adult CECs (p < 0.05, logFC 1). The top 20 regulated genes according to statistical significance are indicated by name (embryonic CECS, n = 5; adult CECs, n = 6).
